# Farnesoid-X receptor as a therapeutic target for inflammatory bowel disease and colorectal cancer

**DOI:** 10.3389/fphar.2022.1016836

**Published:** 2022-10-06

**Authors:** Mengjiao Zhou, Danfeng Wang, Xiang Li, Ying Cao, Chengxue Yi, Dickson Kofi Wiredu Ocansey, Yuling Zhou, Fei Mao

**Affiliations:** ^1^ Key Laboratory of Medical Science and Laboratory Medicine of Jiangsu Province, School of Medicine, Jiangsu University, Zhenjiang, Jiangsu, China; ^2^ Nanjing Jiangning Hospital, Nanjing, Jiangsu, China; ^3^ School of Medical Technology, Zhenjiang College, Zhenjiang, Jiangsu, China; ^4^ Directorate of University Health Services, University of Cape Coast, Cape Coast, Ghana

**Keywords:** Farnesoid X Receptor, immune cells, post-translational modification, inflammatory bowel disease, colorectal cancer, therapy

## Abstract

Farnesoid-X receptor (FXR), as a nuclear receptor activated by bile acids, is a vital molecule involved in bile acid metabolism. Due to its expression in immune cells, FXR has a significant effect on the function of immune cells and the release of chemokines when immune cells sense changes in bile acids. In addition to its regulation by ligands, FXR is also controlled by post-translational modification (PTM) activities such as acetylation, SUMOylation, and methylation. Due to the high expression of FXR in the liver and intestine, it significantly influences intestinal homeostasis under the action of enterohepatic circulation. Thus, FXR protects the intestinal barrier, resists bacterial infection, reduces oxidative stress, inhibits inflammatory reactions, and also acts as a tumor suppressor to impair the multiplication and invasion of tumor cells. These potentials provide new perspectives on the treatment of intestinal conditions, including inflammatory bowel disease (IBD) and its associated colorectal cancer (CRC). Moreover, FXR agonists on the market have certain organizational heterogeneity and may be used in combination with other drugs to achieve a greater therapeutic effect. This review summarizes current data on the role of FXR in bile acid metabolism, regulation of immune cells, and effects of the PTM of FXR. The functions of FXR in intestinal homeostasis and potential application in the treatment of IBD and CRC are discussed.

## Introduction

IBD includes Crohn’s disease (CD) and ulcerative colitis (UC), which over time could progress to CRC (Nadeem et al., 2020). IBD has advanced into a worldwide disease and the prevalence is still increasing, adversely affecting the quality of life of patients (Kaplan, 2015). The pathogenesis involves intestinal ecological environment disorders and autoimmune disorders, and there is no clearly defined etiology. At present, there are many treatment choices, including surgery, medication therapy, fecal microbiota transplantation, etc., but there are still many limitations. For example, fecal microbiota transplantation as a treatment is aimed at *Clostridium difficile*, and the intestinal microbiota is complex, which is easy to cause the recurrence of IBD (Ghouri et al., 2020). Therefore, there is an urgent hope for enhanced beneficial therapeutic methods that can adapt to the complex composition and microbial environment of the intestinal tract.

Different bile acid receptors are activated in the process of bile acid metabolism, including FXR, vitamin D receptor (VDR), pregnane X receptor (PXR), and G protein-coupled bile acid receptor 1 (GPBAR1). FXR is mainly activated by primary bile acids while GPBAR1 is mainly activated by secondary bile acids, and FXR controls the synthesis and secretion of bile acids along with the absorption of bile acids by the gut and liver (Chiang, 2013; Fiorucci et al., 2022). The activation of FXR directly affects bile acid synthesis and the size of the bile acid pool, and its ability to regulate bile acid composition indirectly affects gut microbial diversity, immune response, and the activity of other bile acid receptors (Nie et al., 2015; Fiorucci et al., 2021a). The nuclear FXR was first named in the 1990s due to its activation by the farnesol metabolite. FXR exists in many organs, but primarily in the liver and intestine, and is involved in the regulation of metabolisms. FXR is also expressed in many immune cells such as natural killer T (NKT) cells, mast cells, monocytes, and macrophages (Li et al., 2019; Fiorucci et al., 2022). In response to changes in bile acids, immune cells trigger a series of intracellular FXR activities, therefore, the function of FXR in the immune system is of great value in the study of inflammation and tumor. However, current research mainly focuses on the interaction between FXR and immune cells on liver inflammation and liver cancer. As a vital regulatory receptor of bile acids in the enterohepatic circulation, the interaction between FXR and immune cells in the progression of intestinal inflammation and tumor has not been well explored.

Bile acids could function as endogenous ligands for FXR activation and agonists for FXR have been developed to treat diseases (Anderson and Gayer, 2021). Recently, researchers have turned their attention to the PTM of FXR. An example is the glucose non-oxidant hexosamine biosynthesis pathway that regulates FXR, where the amino-terminal independent transcriptional activation domain 1 (AF1) of FXR interacts with O-GlcNAc transferase and is O-GlcNAcylated. Increased FXR O-GlcNAcylation can subsequently up-regulate FXR gene expression and improve protein stability (Berrabah et al., 2014). Similarly, hepatic FXR phosphorylation at a single residue Tyr-67 is crucial for preventing atherosclerosis in mice (Byun et al., 2019). These indicate that the dysregulation of PTM is closely related to metabolic disorders.

Extensive studies have found that FXR is closely linked to intestinal homeostasis. Abnormal bile acid metabolism gives rise to increased intestinal permeability, causing bacterial translocation (BTL), and FXR protects the intestinal mucosal barrier and prevents bacterial invasion (Verbeke et al., 2015). In addition, FXR is involved in the regulation of oxidative stress and inflammatory response and is connected with cancer-related WNT/β-catenin signaling. Thus, modulating FXR activity has broad therapeutic promise in IBD and related CRC (Yu et al., 2020a; Hua et al., 2021; Miyazaki et al., 2021). Meanwhile, a number of drugs have also been found to regulate FXR and may be used in combination with FXR agonists to compensate for their limitations. This article reviews the structural characteristics of FXR, its metabolic regulation and PTMs, its association with immune cells, the regulation of intestinal homeostasis, and the research progress in IBD and CRC.

## Structural features of FXR

FXR is a type of nuclear receptor activated by bile acid and encoded by the NR1H4 (nuclear receptor subfamily 1, group H, member 4) gene (Keitel et al., 2019; Ramos Pittol et al., 2020). It is distributed in many organs, among which the liver and intestines are most abundant in expression (Wang et al., 2008a). FXR has a typical nuclear receptor structure, including AF1, DNA binding domain (DBD), hinge region, ligand-binding domain (LBD), and carboxy-terminal ligand-dependent transcriptional activation domain 2 (AF2) (Jiang et al., 2021). AF1 and AF2 are the regions of FXR molecules responsible for interacting with regulatory proteins. Ligand-activated FXR connects to the FXR response elements (FXREs) in target genes and can act heterodimers of retinoid X receptor (RXR) or monomers to regulate metabolisms (Stojancevic et al., 2012). FXR commonly exists in the form of FXRα and FXRβ in mammals. FXRα is conserved in humans, encoding four subtypes of FXRα (FXRα 1, FXRα 2, FXRα 3, and FXRα 4), which is the result of diverse uses of promoters and variable splicing of RNA. The expression of FXR is tissue-dependent, with the liver expressing FXRα1/2 at a similar level to FXRα3/4, while the intestine mainly expresses the FXRα3/4 subtypes. Differently, FXRβ is a pseudogene in humans but encodes an available receptor in other species (Wang et al., 2018; Jiang et al., 2021). Due to its characteristics as a ligand-activated transcription factor, explorations of FXR agonists have achieved good results in recent years.

## FXR in bile acid and cholesterol metabolism

It is well known that bile acids are produced by the liver and are reabsorbed in the small intestine through the action of enterohepatic circulation, with approximately 95% of bile acids being reabsorbed in the small intestine (Ocvirk and O'Keefe, 2021; Hofmann, 1984). FXR is the main regulatory factor of bile acid metabolism in the body. Bile acids work as endogenous ligands of FXR, with different capacities of activation, such as chenodeoxycholic acid (CDCA) > deoxycholic acid (DCA) > lithocholic acid (LCA) > cholic acid (CA) (Fiorucci et al., 2021b). FXR controls the size of bile acid pools by changing the vitality of cholesterol 7 alpha-hydroxylase (CYP7A1) to ensure the influx and outflow of bile acids. Moreover, FXR has certain detoxification functions (Kundu et al., 2015). FXR regulates the metabolic homeostasis of bile acids mainly through the hepatic FXR/small heterodimer partner (SHP) axis and the ileal fibroblast growth factor 15/19 (FGF15/19)/hepatic FGF receptor 4 (FGFR4) axis (Fiorucci et al., 2020), which inhibit CYP7A1 activity and thereby inhibit bile acid synthesis (Manley and Ding, 2015; Ticho et al., 2019).

In a study by Kong et al., the effect of FXR deficiency on alcoholic liver disease (ALD) was investigated, reporting that alcohol feeding changed serum and liver bile acid profiles of FXR knockout (FXR-KO) mice. FXR deficiency led to alcoholic hepatitis, increased bile acid synthesis and level, changed bile acid pool composition, and worsened hepatotoxicity (Kong et al., 2019). Since bile acids are made from cholesterol, FXR has been linked to cholesterol metabolism (Makishima, 2005; Chiang, 2013). Moreover, CYP7A1 converts cholesterol into bile acids and is the main way to eliminate cholesterol in the body (Niesor et al., 2001). FXR can control the expression of the CYP7A1 (Cai et al., 2010; Xiao-Rong et al., 2021). It is also reported that there is excessive accumulation of cholesterol in the arterial wall with dysregulation of ceramide metabolism in patients with atherosclerosis (Chambers et al., 2019; Zhang et al., 2019), and sphingomyelin phosphodiesterase 3 (SMPD3) serves as a key enzyme that promotes the sphingomyelin to hydrolyze into ceramide (Liang et al., 2021). Interestingly, SMPD3 has been identified as a target gene for FXR. Intestinal FXR is activated in high-fat-fed mice and the use of intestinal FXR inhibitors could reduce ceramide production and control hypercholesterolemia, leading to new directions in the treatment of atherosclerosis (Wu et al., 2021).

It has been suggested that hepatic and intestinal FXR have different functions in regulating bile acid homeostasis. Intestinal FXR mainly inhibits CYP7A1, while the hepatic FXR mainly inhibits sterol 12 alpha-hydroxylase (CYP8B1), and the inhibitory effect on CYP7A1 is far less than that of intestinal FXR. The main reason is the effect of intestinal FXR-FGF15 signal. Under the action of FXR agonist GW4064, FGF15 is almost not induced in the ileum of mice with intestine-specific FXR deficiency, but the expression of FGF15 is higher in the ileum of mice with liver-specific FXR deficiency. GW4064 significantly inhibited CYP7A1 expression in liver FXR-specific knockout mice but had no significant effect on intestine FXR-specific knockout mice. At the same time, the loss of FGF15 caused GW4064 to significantly inhibit the level of CYP8B1 but had no significant effect on the level of CYP7A1. The fecal DCA content of mice with intestinal FXR-specific deficiency was also much higher than that of mice with liver-specific deficiency of FXR (Kim et al., 2007). Similarly, Modica et al. also found that selective activation of intestinal FXR could induce FXR-FGF15 signaling, and then reduce the total bile acid pool, which is sufficient to relieve the symptoms of cholecystitis and intestinal mucosal damage (Modica et al., 2012). To some extent, hepatic FXR and intestinal FXR have complementary effects on the inhibition of bile acid synthesis. The effects of hepatic FXR are mainly focused on the protection of cholesterol homeostasis and associated lipid accumulation levels. Mice with liver-specific FXR deficiency have higher serum triglyceride and cholesterol levels than mice with intestine-specific FXR deficiency. Moreover, there is more lipid accumulation in liver tissue sections, and specific deficiency of liver FXR induced high levels of the liver X receptor (LXR), however, this effect was not related to intestinal FXR-FGF15 signaling (Schmitt et al., 2015).

## FXR and immune cells

Not only can FXR take part in the regulation of metabolism, but also the modulation of immune cells. Changes in bile acid metabolism affect the transformation of immune cytokines and the impacts on immune cells. Therefore, as a key regulatory receptor of bile acid metabolism, FXR plays a crucial regulatory role in the immune system. Studies in this area have focused on both direct effects due to the activation of FXR and indirect effects that are related to changes in bile acid pool composition.

## T cells and NKT cells

While changes in bile acid metabolism affect T cells, T cells can also directly affect changes in the bile acid spectrum. Cholangitis induced by the adoptive infusion of CD8^+^T cells into the liver inhibits the production of unbound bile acids, and upregulates the level of bound bile acids, but does not lead to hepatocyte apoptosis. Therefore, to some extent, T cells do not produce harmful bile acid metabolism. However, this process requires the involvement of the proinflammatory factors tumor necrosis factor (TNF) and interferon-γ (IFN-γ), both of which depend on changes in hepatic FXR levels (Glaser et al., 2019). Except for the uncommon NKT cells, there is currently no evidence that T cells themselves express FXR (Fiorucci et al., 2022). An abnormal bile acid signal is also a promoter of tumorigenesis. As one of the components of bile acid, Nor Cholic acid (NorCA) can promote the immune escape of liver cancer cells and obviously increase Programmed Cell Death-1 (PD-1) on the surface of CD4^+^ T cells by destroying the FXR/SHP signal axis, accompanied by upregulation of Programmed Cell Death Ligand-1 (PD-L1) in hepatocellular carcinoma (HCC) cells and their secreted exosomes. It is well documented that PD-1/PD-L1 compromises T cell activity, which is related to the immune escape of tumors. However, under the treatment with the FXR agonist GW4064, the levels of PD-1 and PD-L1 are significantly inhibited. Gong et al. used a combination of FXR agonist and anti-PD-1 antibody to highlight insights into the use of the tumor immune microenvironment against HCC for the treatment of other tumors (Gong et al., 2021). Under inflammatory conditions, effector T cells can be recruited to the intestinal tract through the α4β7-Madcam1 axis to promote the inflammatory process. Under the action of obeticholic acid (OCA), the expression of chemokine Madcam1 can be significantly inhibited, thereby reducing the aggregation of effector T cells to the intestinal tract, while regulatory T cells (Tregs) rely on the CCL25-CCR9 axis to home to the intestine to play their role in inhibiting inflammation (Massafra et al., 2016). In another study, CDCA promoted the expression of FXR in an acute murine asthma model and inhibited the production of T-helper (Th) 2 cytokines, mainly interleukin (IL)-4, IL-5, and IL-13, and decreased the level of immunoglobulin E (IgE) in the lung. Thus, FXR induces the function of T cytokines and is also a lifesaving mechanism against respiratory inflammatory diseases (Shaik et al., 2015).

NKT cells are a special subset of T cells that express both NK cell receptors and T cell receptors (Cui and Wan, 2019). Triptolide (TP), an immunosuppressant, stimulates invariant Natural Killer T (iNKT) cells to secrete Th 2 cytokines, and at the same time, interacts with type 2 NKT cells to inhibit their protective effect on the liver. FXR expression is inhibited by TP at both the transcriptional and translational levels. However, the knockdown of iNKT cells alleviates liver injury and up-regulates FXR expression. Therefore, the combination of TP and FXR agonists may alleviate liver injury caused by TP (Zou et al., 2020). Considering the potential of FXR agonists, scholars are exploring how to efficiently absorb them during their application. Ji et al. created OCA-nanoemulsion (OCA-NE) to better attach OCA to the liver, which more accurately promotes the secretion of CXC chemokine ligand 16 (CXCL16) by liver sinusoidal endothelial cells (LSECs) compared with the traditional oral OCA. In turn, NKT cells are triggered to accumulate in the tumor site to exert an anti-tumor immune effect (Ji et al., 2020). Moreover, NKT cells themselves also express FXR. High expression of osteopontin (OPN) in NKT cells induced by Concanavalin A (ConA) can promote the expression of glycosylated protein OPN in liver inflammation. However, under the action of 6alpha-ethyl-chenodeoxycholic acid (6-ECDCA), also known as INT-747 and OCA (Pellicciari et al., 2002; Fiorucci et al., 2022), the FXR/SHP signaling axis is effectively activated and the development of inflammation is inhibited (Mencarelli et al., 2009).

## Macrophages and monocytes

Macrophages play a momentous role in various immune diseases, M1 and M2 are different subtypes that adapt to environmental changes. M1 macrophage plays a role in promoting inflammation and its main markers include CD80, CD86, and inducible nitric oxide synthase (iNOS), while M2 type plays an antagonistic role in inflammation, and its main markers are arginase 1 (Arg1), CD206, and CD163 (Kadomoto et al., 2021). Yao et al. found that the activation of FXR with GW4064 alleviates liver injury caused by lipopolysaccharide (LPS) by disturbing the release of inflammatory cytokines from macrophages in the non-alcoholic fatty liver disease (NAFLD) mice model. In addition, the apoptosis of liver cells was observably reduced and the infiltration of macrophages with the F4/80 marker in liver tissue was also suppressed (Yao et al., 2014). Interestingly, Cao et al. reached similar conclusions in chronic periaortitis, an autoimmune disease distinguished by fibrosis and increased inflammatory response. CDCA, as a natural agonist of FXR and a component of bile acids, has a defensive increase in chronic periaortitis patients to promote the nuclear translocation of FXR in macrophages and inhibit the production of IL-6, a pro-inflammatory and pro-fibrotic factor, although CDCA does not add to the overall expression of FXR in macrophages (Cao et al., 2021). Macrophages are the main source of monocyte chemoattractant protein-1 (MCP-1) which promotes the infiltration and invasion of macrophages to the inflammatory site, while CDCA can inhibit the expression of MCP-1. Mechanistically, CDCA promotes FXR to stick to a DR4 component in the promoter region of MCP-1, thereby inhibiting the expression of MCP-1 (Li et al., 2015). Therefore, it can be speculated that the CDCA-FXR axis works as a good modulator of the anti-inflammatory function of macrophages.

In the LPS-induced model of macrophage inflammation, SUMOylation of FXR in macrophages is inhibited and INT-747 could promote SUMOylation of FXR and stabilize the nuclear co-repressor (NCoR) at the promoters of iNOS and IL-1β, effectively inhibiting inflammation (Vavassori et al., 2009). In the study of Hao et al., it was found that bile acid metabolism was abnormal in patients with cholestasis. High levels of bile acids could act as damage-associated molecular patterns (DAMPs) to promote NOD-like receptor thermal protein domain associated protein 3 (NLRP3) inflammasome activation in macrophages, while FXR inhibits NLRP3 inflammasome activity. Primary macrophages isolated from FXR-KO mice also showed spontaneous NLRP3 activation compared with wild-type (WT) mice. The main reason is that FXR can directly interact with NLRP3 or caspase 1 physically, without the bridge of promoting FXR transcription in a ligand-dependent manner, so the presence of FXR plays an important anti-inflammatory role in macrophages (Hao et al., 2017). However, FXR can also inhibit the activation of NLRP3 inflammasome through an SHP-dependent pathway. In the study of Yang et al., SHP in macrophages can directly act on NLRP3 to inhibit the formation of NLRP3 and apoptosis-associated speck-like protein containing a caspase recruit domain (ASC) complex, thereby inhibiting the activation of NLRP3 inflammasome and reducing mitochondrial damage to relieve inflammation (Yang et al., 2015).

Monocytes include two subsets, Ly6C^low^ and Ly6C^high^. Ly6C^low^ monocytes suppress inflammation, while Ly6C^high^ monocytes promote inflammation. There is evidence of the ability of monocytes to express FXR (Fiorucci et al., 2022). Dual FXR/GPBAR1 agonist (INT-767) significantly inhibits the development of liver inflammation and fibrosis in NAFLD mice, and the mechanism is that INT-767 promotes the increase of Ly6C^low^ monocytes and decrease of Ly6C^high^ monocytes by directly targeting Ly6C levels. INT-767 also promotes the differentiation of monocytes into M2 macrophages (McMahan et al., 2013). These promising observations require that the specific molecular mechanism of INT-767 regulating FXR in monocytes be further elaborated in future studies. Chronic inflammation in conditions such as HIV/AIDS continues to be of major concern. Data indicates that the expression of nuclear receptors such as FXR, PXR, and RXR in monocytes of patients with HIV infection is significantly decreased. The highly active antiretroviral therapy (HAART) can only inhibit the replication of the virus but cannot restore the expression of nuclear receptors, and the development of chronic inflammation still persists. The combination of HAART and FXR agonists may inhibit both inflammatory responses and viral replication (Renga et al., 2012).

## Mast cells

**FIGURE 1:**
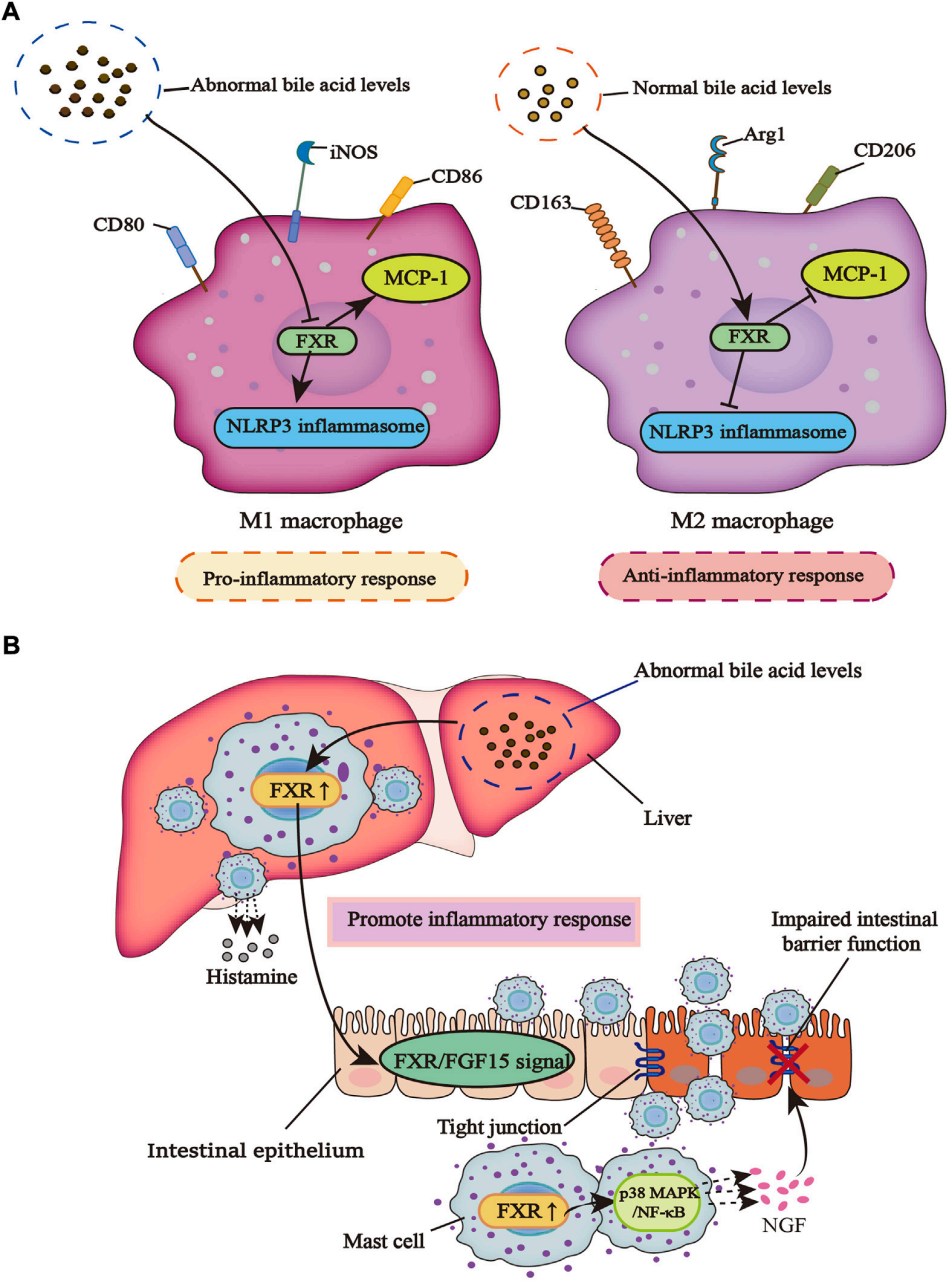
The different effects of FXR on macrophages and mast cells in an inflammatory response. **(A)** When bile acid metabolism is abnormal, macrophages are induced to the M1 type, which can promote inflammation and inhibit the regulation of FXR on NLRP3 inflammasome and MCP-1. However, in a normal bile acid metabolism, macrophages are polarized into the M2 type, which can inhibit inflammation and promote the regulation of FXR on NLRP3 inflammasome and MCP-1. **(B)** When bile acid metabolism is dysregulated, the infiltration of liver mast cells increases along with the increased release of histamine, which can promote the activity of FXR in liver mast cells. This leads to the activation of intestinal FXR/FGF15 signal under the action of enterohepatic circulation, and the infiltration of intestinal mast cells also increases. Activated FXR promotes the P38 MAPK/NF-κB signaling pathway, causing MCs to release more NGF, a mediator that can damage tight junctions in the gut, leading to a breakdown of the intestinal barrier and increased inflammatory response.

Mast cells (MCs), which are important innate immune cells, degranulate in response to stimuli (Espinosa and Valitutti, 2018). After degranulation, the bioactive ingredients produced by MCs affect inflammation, fibrosis, and even tumorigenesis (Mukai et al., 2018). In diarrhea irritable bowel syndrome (IBS-D) patients with high levels of total bile acids in the colonic mucosa that leads to visceral hypersensitivity (VH), Li et al. found that under the stimulation of bile acids, mucosal mast cells (MMCs) secrete nerve growth factor (NGF), a mediator that can damage colonic tight junctions. It is through the NGF/transient receptor potential vanillin (TRPV)1 axis that VH is produced. However, there is no bile acids-induced overexpression of NGF in FXR-KO mice. At the same time, FXR is expressed in MCs and regulates the level of NGF expressed by MMCs through regulating its downstream P38 mitogen-activated protein kinases (MAPK)/nuclear factor κB (NF-κB) signaling pathway (Li et al., 2019). The progress of primary sclerosing cholangitis (PSC) is often accompanied by the emergence of IBD. There is increased infiltration of hepatic MCs in cholestasis and the high level of MCs-FXR crosstalk with intestinal FXR/FGF15 signal induces the infiltration of intestinal MCs, which leads to the increase of inflammatory response and promotes the transduction of serum histamine (HA) signal. The inhibition of MC-FXR significantly improves the level of total bile acids and inhibits inflammation. Contrary to these observations, previous studies reported that high levels of FXR in MCs can lead to a harmful outcome, which may be related to the different effects of FXR on different types of immune cells (Meadows et al., 2021) (Figure 1).

FXR agonists are now being considered as a treatment option for many diseases, and it is worth noting that their effects on immune cells vary between diseases (Table 1). And the FXR agonist, OCA, was originally discovered by Pellicciari and colleagues reported as 6-ECDCA (Pellicciari et al., 2002), and then christened by the same group as INT-747 and later renamed as OCA (Fiorucci et al., 2022). These different names have been used in different studies exploring the effects of FXR in different models of diseases.

**TABLE 1:** Effects of FXR agonists on immune cells in diseases.

FXR agonist	Disease	Immune cells	Effect of FXR agonists	Reference
OCA (INT-747; 6-ECDCA)	Acute hepatitis	NKT cells	OPN from NKT cells ↓	Mencarelli et al., 2009
			Liver injury ↓	(Mencarelli et al., 2009)
			Inflammation ↓	
	Colitis	Macrophages	FXR SUMOylation ↑	Vavassori et al., 2009
			Stabilize NCoR on the promoters of iNOS and IL-1β	(Vavassori et al., 2009)
			TNF-α ↓	
			Inflammation ↓	
	IBD	Dendritic cells (DCs),	Splenic DCs ↑	Massafra et al., 2016
		effector T cells and Tregs	Migration of DCs and effector T cells to the colon ↓	(Massafra et al., 2016)
			Migration of Tregs to the colon ↑	
			IL-10 ↑	
			Colonic inflammation ↓	
	HCC	NKT cells	Numbers of NKT cells ↑	Ji et al., 2020 (Ji et al., 2020)
			Accumulation of NKT cells in the tumor ↑	
			Anti-tumor ability ↑	
GW4064	Immune-mediated hepatitis	Myeloid-derived sup-Pressor cells (MDSCs)	Numbers of MDSCs ↑	Zhang et al., 2014 (Zhang et al., 2014)
			Expression of paired immunoglobin-like receptor-B (PIR-B) in MDSCs ↑	
			The immunosuppressive activity of MDSCs ↑	
			Liver injury↓	
	Experimental autoimmune encephalomyelitis (EAE)	Macrophages	Anti-inflammatory macrophages ↑	Hucke et al., 2016 (Hucke et al., 2016)
			IL-10 ↑	
			Improve central nervous system autoimmunity	
	HCC	CD4^+^ T cell	PD-1 from CD4^+^ T cell ↓	Gong et al., 2021 (Gong et al., 2021)
			The function of CD4^+^ T cells ↑	
			Anti-tumor ability ↑	
	Chronic periaortitis	Macrophages	IL-6 produced by macrophages ↓	Cao et al., 2021 (Cao et al., 2021)
			Transportation of FXR into the nucleus ↑	
			Inflammation ↓	
			Fibrosis ↓	
INT-767	NAFLD	Macrophages and Monocytes	Numbers of Ly6C^low^ monocytes ↑	McMahan et al., 2013 (McMahan et al., 2013)
			Numbers of Ly6C^high^ monocytes ↓	
			Ly6C levels of monocytes ↓	
			Numbers of M2 macrophages↑	
			Inflammation ↓	
CDCA	Allergic asthma	Th2 cells	Serum IgE ↓	Shaik et al., 2015 (Shaik et al., 2015)
			Th2 cytokines (IL-4, IL-5 and IL-13) ↓	
			Airway inflammation ↓	
	VH	MCs	NGF expression in MCs ↑ p38 MAPK/NF-κB signaling ↑	Li et al., 2019 (Li et al., 2019)
			Damage to the colonic tight junction	

## Post-translational modifications of FXR

FXR can affect a myriad of physiological processes, including homeostasis and metabolism. Many studies prove that PTMs of FXR have an impact on the mechanism of action of diseases. PTM is crucial in regulating protein folding, activity, and function, allowing proteins to have multiple structures, perform more complex functions, and perform different tasks with greater precision, such as acetylation, SUMOylation, and methylation (He et al., 2018). Previous studies on the epigenetic modification of FXR could provide innovative ideas about diseases (Anbalagan et al., 2012; Wan and Sheng, 2018).

## Acetylation

Using tandem mass spectrometry (MS/MS), Kim et al. found that the FXR hinge region, lysine (K)217 is the main acetylation site and also observed the acetylation of the DNA binding region K157. P300 and sirtuin 1(SIRT1) strictly adjust the acetylation of FXR, and P300 makes FXR acetylation. By employing molecular, cellular, and animal studies, it was confirmed that FXR is the regulatory target of SIRT1, SIRT1 has direct interaction with FXR and carries out FXR deacetylation, a process capable of destroying the stability of FXR and rendering it more easily degraded. When FXR is acetylated, it decreases the expression of FXR, impairs the heterodimerization of FXR/RXR with DNA, and reduces the trans-activation ability of FXR. Some scholars report that the levels of FXR acetylation are evidently higher in mice fed with a western-style fat diet for a long time than in mice with a normal diet. Thus, excessive acetylation of FXR results in harmful metabolism, which in turn harms health (Kemper et al., 2009). Resveratrol (RSV), a natural phenolic compound produced by plants, is a SIRT1 activator in nature (Deng et al., 2021), and reduces oxidative stress and prevents the production of pro-inflammatory factors by improving the antioxidant defense system. It was found that the reduced expressions of SIRT1, LXR, and FXR were associated with increased levels of lipid, alanine transaminase (ALT), alkaline phosphatase (ALP), and aspartate transaminase (AST) in rats with NAFLD, along with a significantly increased percentage of hepatocyte apoptosis (Hajighasem et al., 2013). The administration of RSV activates SIRT1, LXR, and FXR, and effectively improves abnormal lipid levels and hepatocyte apoptosis. As SIRT1 is an NAD^+^ histone deacetylase (HDAC)-dependent enzyme, it is strongly associated with gene regulation, apoptosis, autophagy, inflammation, and tumor occurrence (Alves-Fernandes and Jasiulionis, 2019). Therefore, making good use of SIRT1-FXR as a signal axis can widely regulate a variety of inflammatory and metabolic diseases (Kulkarni et al., 2016; Sun et al., 2020; Ma et al., 2021).

## SUMOylation

There are three subtypes of small ubiquitin-like modifiers (SUMO) protein in mammals: SUMO-1, SUMO-2, and SUMO-3. SUMO-1 primarily embellishes physiological state proteins, while SUMO-2 embellishes stress proteins. SUMOylation is essential for the regulation of gene expression and the control of intracellular signal transduction since this pathway exists in almost all eukaryotes (Han et al., 2018). In their *in vitro* and *in vivo* experiments, Luo et al. found that FXR could be SUMOylated and therefore predicted that FXR SUMOylation might play a role in regulating liver function. There are several classic SUMO consensus sites in the AF-1 and LBD of FXR, and the sequential chromatin immunoprecipitation (ChIP-reChIP) confirmed the simultaneous binding of FXR and SUMO-1 to the bile salt export pump (BSEP) and SHP promoters, which are target genes of FXR. The overexpression of SUMO-1 significantly reduces the connection of FXR to SHP and BSEP promoters. Similarly, in HepG2 cells, small interfering RNA (siRNA) knockout of SUMO-1 increases the trans-activation of BSEP and SHP promoters. At the same time, the main attachment sites for SUMO-1 are K122 and 275 of FXR since K122 and 275 constrict SUMOylation consensus sites when mutated, eliminating FXR SUMOylation. It is worth noting that FXR SUMOylation is specific, reversible, and highly dynamic (Balasubramaniyan et al., 2013). Proteomic studies have shown that diet-induced acetylation of FXR at K217 occurs in mice. For example, 8–12 weeks of a high-fat diet (HFD) augmented the acetylation level of FXR but lessened the SUMO-2 level of FXR, causing a significantly activated expression of inflammatory genes. Interestingly, the SUMOylation of FXR promoted by GW4064 enhanced its connection with NF-κB. Selective restraint of inflammatory genes instead of influencing the expression of target genes of FXR/RXR demonstrates the anti-inflammatory effect of SUMO-FXR. Dysregulation of the FXR acetyl/SUMO switch may be the general mechanism by which anti-inflammatory responses of other transcriptional regulators are reduced, and may supply potential salutary and diagnostic methods for IBD and some inflammatory-related metabolic illnesses (Kim et al., 2015). Importantly, as early as 2009, Vavassori et al. found that FXR SUMOylation decreased in the model of macrophage inflammation induced by LPS, while FXR agonist INT-747 significantly promoted FXR SUMOylation and effectively inhibited the development of inflammation (Vavassori et al., 2009).

Interestingly, Gao et al. demonstrated that myocardial ischemia/reperfusion (MI/R) injury leads to massive apoptosis and that the levels of FXR SUMOylation play a role in regulating the apoptosis of cardiomyocytes. The SUMOylation of FXR occurs in normal heart tissues, and the SUMOylation of MI/R decreases, activating the mitochondrial apoptosis pathway (Gao et al., 2018). These results make clear that FXR SUMOylation also has great significance in apoptosis.

## Methylation

Methylation is a vital modification of protein and nucleic acid, which goes hand in hand with many diseases such as cancer, senile dementia, and aging. The most common methylation modifications are DNA methylation and histone methylation. Intrahepatic cholestasis of pregnancy (ICP) is known as harmful bile acid metabolism during pregnancy, but symptoms quickly resolve after the end of pregnancy (Keitel et al., 2016). Therefore, some scholars believe that ICP pathogenesis may be deeply regulated by epigenetics. Based on samples from 88 ICP patients and 173 normal pregnant women in the third trimester, researchers showed that CpG dinucleotide of promoters of nuclear receptor subfamily 1 gene and adenosine triphosphate (ATP) binding box transporters were highly methylated during normal pregnancy, while methylation levels were decreased in ICP patients, and the methylation levels also influenced bile acid profiles. At the same time, the methylation of the distal and proximal promoters of the nuclear receptor FXR/NR1H4 had different effects. Since the distal promoters bind to transcription factors in a methylation-dependent manner and transcriptional regulatory elements can act as enhancers, they are more related to gene expression in the whole genome (Cabrerizo et al., 2014). Ann M. Bailey et al. found reduced FXR expression in early colon cancer owing to DNA methylation of the FXR promoter and enhanced Kirsten rat sarcoma viral oncogene (KRAS) signaling. DNA methylation is one mechanism that results in FXR silencing in colon cancer (Bailey et al., 2014). In addition, since BSEP is the target gene of FXR, FXR has histone methyltransferase activity at its locus, which is specific to arginine 17 of histone H3 and regulates BSEP expression (Ananthanarayanan et al., 2004).

In addition, the role of FXR phosphorylation and glycosylation has also been reported (Berrabah et al., 2014; Byun et al., 2019). The activity of FXR is mainly controlled by the ligand it binds to, but the PTM of FXR is another important factor, and PTM may be more influential than bile acid flux during feeding and fasting cycles (Panzitt and Wagner, 2021) (Table 2). At present, there is still little and limited research on the regulation of FXR and its target genes from the perspective of epigenetic modification. Perhaps, more discoveries can be made by focusing on this foothold.

**TABLE 2:** The post-translational modifications of FXR.

Post-translational modification	Site	Influence	Model	Reference
Acetylation	K217 and K157	Suppress FXR/RXRα heterodimerization and promote the stability of FXR.	Obesity and type II diabetes mice	Kemper et al. (2009)
Methylation	K206	Methylation by Set7/9 strengthens the binding of FXR/RXRα to the FXRE and then activates the transcription of FXR target genes	Huh-7 liver cell line and CV-1 monkey kidney line	Balasubramaniyan et al. (2012)
O-GLcNAcylation	S (serine) 62	Increase FXR protein stability and transcriptional activity through SMRT-containing corepressor complexes inactivation	C57Bl6/J male mice	(Benhamed et al., 2014; Berrabah et al., 2014; Appelman et al., 2021)
Phosphorylation	Tyr-67	Promote FGF19-mediated cholesterol efflux from hepatocytes and reduce plasma cholesterol to relieve atherosclerosis	FXR-floxed mice	Byun et al. (2019)
SUMOylation	K122 and K275	Inhibit the combination of FXR at the promoters of its target genes, probably by disrupting the binding with FXRE or preventing FXR from dimerizing with RXRα	HepG2 liver cell line and the CV-1 monkey kidney cell line	Balasubramaniyan et al. (2013)
	K277	SUMOylation of FXR induced by INT-747 stabilizes the NCoR on the promoters of iNOS and IL-1β to inhibit inflammation	THP-1 cells	Vavassori et al. (2009)

## FXR and intestinal homeostasis

FXR is the main regulatory factor of the entire hepatoenteric axis. Previous research mainly concentrated on the role of FXR and FXR agonists in liver diseases, but with the deepening of research, it has been found that FXR also shines in intestinal homeostasis, effectively protecting the intestinal mucosa, and plays a certain antibacterial role. The reports of a real-time quantitative PCR (RTQ-PCR) analysis indicated that FXR is mainly expressed in the ileum, colon, duodenum, and jejunum. Besides, FXR is not only highly expressed in the gut, but also has relative target genes that regulate it, including the SHP, ileal bile acid-binding protein (IBABP), and FGF15/19. However, there are many unknowns concerning how target genes are regulated and the mechanisms of action involved in intestinal disease (Inagaki et al., 2006). Under normal circumstances, there are a lot of bacteria in the intestinal tract, which restrict and depend on each other, forming a complex ecosystem. A study found that the number of aerobic bacteria in mesenteric lymph nodes of FXR-KO mice was 10 times more than that in WT mice (Inagaki et al., 2006). The excessive accumulation of the bile acids caused intestinal mucosal damage and bacterial proliferation, generating intestinal bacterial translocation, which is the transfer of bacteria from the intestinal lumen to lymph nodes of the mesentery and subsequently to external parts such as the peritoneum. In several cases, it can cause multiple organ failures and even death, while at the same time, the intestinal permeability can be increased and the intestinal epithelial barrier can be destroyed. In effect, FXR deficiency is associated with increased intestinal permeability, which then causes bacterial translocation. Moreover, the inactivation of intestinal FXR occurs in the rat’s model of cholestasis. The use of FXR agonist INT-747 restores intestinal permeability, promotes the expression of tight junction proteins claudin-1 and occludin in the ileum of rats, and promotes intestinal and systemic anti-inflammatory responses (Verbeke et al., 2015; Miyazaki et al., 2021).

Inflammasome activation plays a vital role in the immune defense of hosts against bacterial infection and is an important part of the natural immune system. Inflammasomes can discern pathogen-associated molecular patterns (PAMPs) or DAMPs to recruit and activate the pro-inflammatory protease caspase-1, where caspase-1 subsequently cleaves the precursors of IL-1β and IL-18, promoting the secretion of mature cytokines IL-1β and IL-18, a process valuable to intestinal homeostasis (Zhen and Zhang, 2019). Kang et al. demonstrated that the abnormal inflammasome activation in FXR-deficient mouse primary macrophage (BMDM) is associated with reduced IL-1β expression. After infection with *Listeria monocytogenes* or *Escherichia coli*, FXR-deficient BMDM showed decreased caspase-1 (P20) and caspase-11 (P30) activity compared to WT cells. The release of IL-1β was also observably lower than WT cells by enzyme-linked immunosorbent assay (ELISA). FXR-deficient mice were unable to effectively eliminate bacteria and had higher mortality and bacterial load than WT mice. Treatment with 6-ECDCA reduced bacterial load *in vitro* and increased survival *in vivo*. However, an interesting fact is that NLRP3 deficiency did not eliminate 6-ECDCA-induced bacterial clearance. Regardless, FXR could promote the inflammasome-mediated antibacterial response to a certain extent and may become a new target of antibacterial therapy (Kang et al., 2021). In a similar study, Zahiri et al. found that taurodeoxycholate (TDCA) supplementation promotes cell renewal to assist the maintenance of mucosal epithelium integrity and increases the expression of c-Myc, a key regulator of cell propagation, by increasing the expression of FXR. In addition, the intestinal villi length of mice treated with endotoxin was significantly shortened, but after TDCA supplementation, functional FXR maintained intestinal villi height compared with FXR-KO mice (Zahiri et al., 2011; Luo et al., 2019). Therefore, many FXR agonists have been developed to improve the activity of FXR and have been used to better execute the anti-inflammatory and antioxidant effects of FXR.

On the other hand, overactivation of FXR can sometimes lead to a negative outcome. Paneth cells are key elements of the intestinal stem cell (ISC) microenvironment (Schoenborn et al., 2019; Hou et al., 2020), generating antimicrobial peptides to protect the intestinal barrier (Battistini et al., 2020), and are usually found in the cecum and ascending colon (Yu et al., 2020b). Surprisingly, in western-diet-fed mice, overactivation of FXR was found to lead to defects in Paneth cells due to increased secondary bile acids (specifically DCA) in the ileum. Possible reasons for this observation include FXR overactivation directly affecting Paneth cells and FXR activation increasing the production of type I interferon in intestinal myeloid cells, resulting in abnormal Paneth cells (Liu et al., 2021). It can be seen that the regulatory mechanism of FXR in the intestinal tract is complicated to some extent, but in general, improving the activity of FXR must be more beneficial than harmful to intestinal diseases. In the regulation of intestinal homeostasis, FXR is still a new star worth exploring and full of potential.

## The progress of FXR in IBD

The bile acid signal in IBD patients is significantly different from that of healthy people. The serum and fecal secondary bile acid levels are decreased while fecal-bound bile acid excretion is increased (Baars et al., 2015). This is closely related to the complex intestinal microbiota environment, as primary bile acids are modified into secondary bile acids by the intestinal microbiota. Studies indicate that the diversity of intestinal microbiota in IBD patients is decreased, with a characteristic reduction in firmicutes and elevation of proteobacteria. Since firmicutes are the main force of bile acid modification, their significant reduction in IBD patients affects the bile acid pattern. The protective effect of secondary bile acids on the gut decreases while primary bile acids increase (Franzosa et al., 2019; Li et al., 2021). In addition, a study found that the abundance of firmicutes and their bile salt biotransformation genes (BSBGs) were dominant in normal human and IBD samples in bioinformatics analysis of secondary bile acid metabolites, and the reduction of firmicutes in IBD was related to the reduced capacity of biliary salt transformation (Das et al., 2019). Since the ileum is primarily responsible for the reabsorption of conjugated bile acid, patients with CD often have bile acid malabsorption due to ileum dysfunction, which leads to a smaller pool of bile acids (Pavlidis et al., 2015; Vítek, 2015). The dysregulation of bile acid metabolism damages intestinal mucosa and promotes the inflammatory response. FXR serves as a key receptor regulating bile acid synthesis and plays an important role in IBD.

**FIGURE 2:**
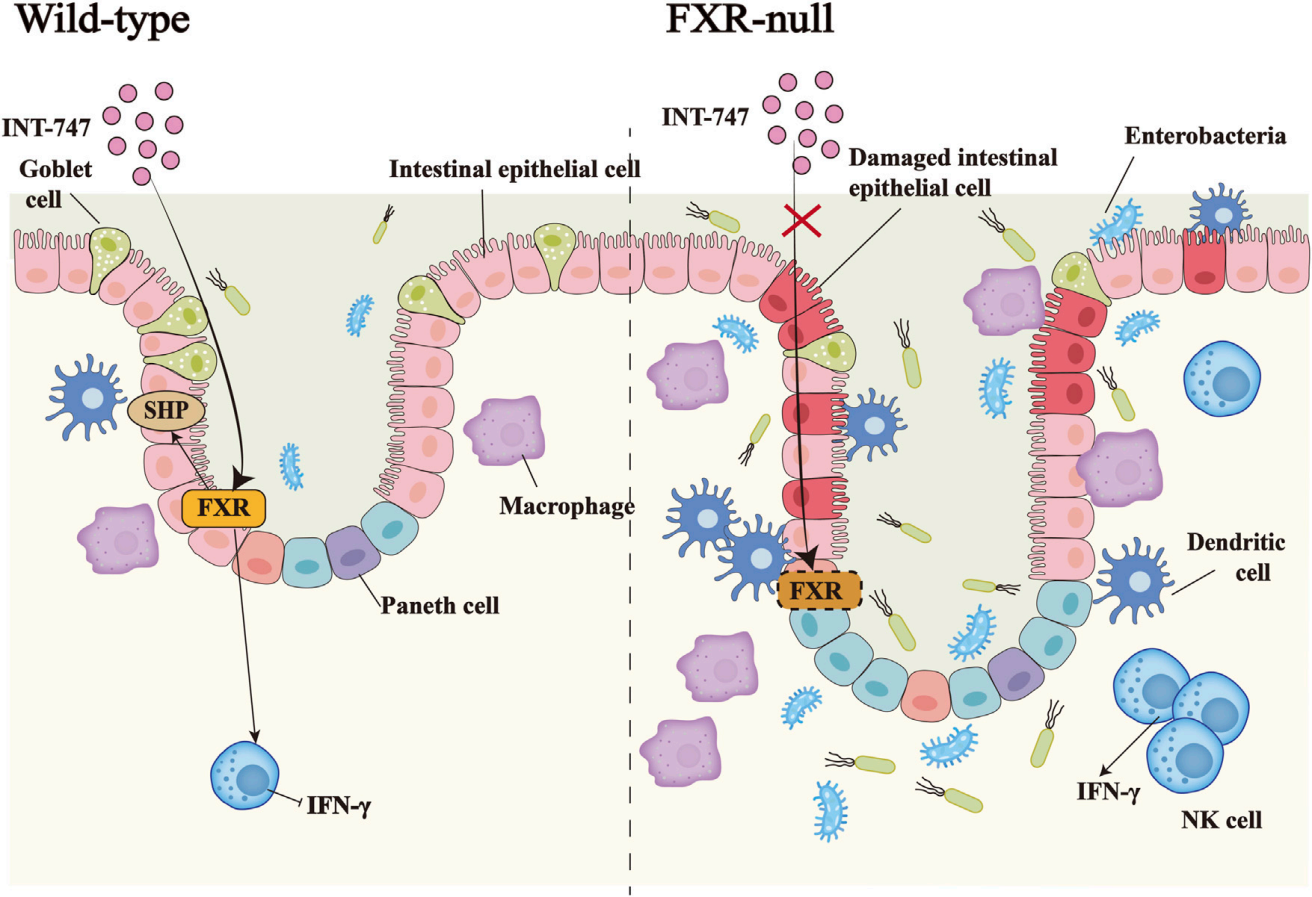
The importance of FXR in inflammatory bowel disease. In DSS-induced IBD, the administration of the FXR agonist INT-747 to FXR-KO mice and WT mice, activates the expression of FXR in the intestinal tract of WT mice, thereby promoting the upregulation of the target gene SHP of FXR and inhibiting the release of the pro-inflammatory factor IFN-γ by NK cells. INT-747 reduced intestinal permeability in WT mice, while FXR-KO mice could not have the same phenomenon due to the loss of the FXR gene. Meanwhile, FXR-KO mice had more damaged intestinal epithelial cells, fewer goblet cells, and increased bacterial invasion. The number of dendritic cells, macrophages, NK cells, and other immune cells also increased, which led to susceptibility to bacterial translocations.

Bile acids pass through the intestine of the healthy person 6–10 times a day in response to the enterohepatic circulation. Repeated stimulation of the intestinal mucosa by hydrophobic bile acids can trigger intestinal inflammation, leading to cell death and apoptosis, and FXR can strictly control the homeostasis of bile acids in the human body to prevent their concentration from reaching cytotoxic levels (Ibrahim et al., 2019; Engin, 2021). Decreased intestinal FXR activity has been found in IBD patients and FXR mRNA expression is also reduced in inflamed colonic mucosa (Baars et al., 2015; Wilson et al., 2020). In our recent studies, we reported downregulated expression of FXR in both IBD patients and dextran sodium sulfate (DSS)-induced IBD mice model, which was accompanied by altered primary bile acid biosynthesis. Treatment of the animal model with mesenchymal stem cell-derived exosomes significantly restored colonic FXR alongside improved gut microbiota metagenomics and metabolomics, resulting in relieving macroscopic and microscopic features of IBD (Ocansey et al., 2022; Xu et al., 2022). In the mice model of colitis, the intestines of FXR-deficient mice show a more severe proinflammatory and profibrotic state, accompanied by immune dysfunction. INT-747 treatment could inhibit colitis effectively in WT mice, but not in FXR-deficient mice (Vavassori et al., 2009). The FXR agonist INT-747 also has been found to significantly reduce weight loss and prevent colon shortening in WT mice, meanwhile, FXR activation partially reduces goblet cell loss, protects the intestinal barrier, and inhibits inflammatory response (Gadaleta et al., 2011a) (Figure 2). In patients with IBD, high promotion of NF-κB is a key link to the inflammatory response, giving rise to strongly increased generation of pro-inflammatory cytokines (TNF-α, IL-8, and IL-6) and nitric oxide (NO) production. This is due to excessive activation of NF-κB subunits P50 and P65, which leads to FXR inhibition (Gadaleta et al., 2011b). In addition, target genes of FXR such as SHP, IBABP, and FGF15/19 are suppressed through reduced FXR (Wang et al., 2008b; Liu et al., 2016; Guo et al., 2021). Simultaneously, conservative NF-κB binding sites are also found in the promoter of the FXR target gene (Balasubramaniyan et al., 2016).

Liu et al. constructed a model of IBD in WT and FXR KO mice by injecting LPS. Results showed that GW4064 inhibited LPS-induced toll-like receptor 4 (TLR4)/myeloid differentiation primary response 88 (MyD88) pathway and increased the levels of FXR protein and SHP mRNA. This relieved the breakdown of tight junction function caused by LPS and reduced macrophage infiltration in WT mice but had less impact in FXR KO mice. In other words, LPS resulted in a dramatically increased expression of TLR4 and MYD88 in WT and FXR KO mice, while treatment with GW4064 significantly reduced TLR4 and MYD88 protein levels in WT mice but did not significantly change in FXR KO mice. Similarly, LPS induced a reduced expression of zonula occludens-1 and claudin-1 in FXR KO mice compared to WT mice, while GW4064 markedly elevated their expression in WT mice, with little effect on FXR KO mice. By analyzing the markers F4/80 and CD11b of the ileum lamina proper macrophages, LPS upregulated F4/80 and CD11b in WT mice, while GW4064 treatment completely inhibited this effect. The markers F4/80 and CD11b in FXR KO mice were more highly expressed than in WT mice and showed an upward trend, however, GW4064 treatment could not reverse this outcome in FXR KO mice (Liu et al., 2017).

Some studies have reported that since the intestine and liver are connected through portal vein circulation, the regulatory function of bile acid is mainly mediated by bile acid receptors, and they can reduce intestinal and liver inflammation. By studying the liver of a 3% DSS-induced colitis model of mice, it was found that FXR and PXR were significantly down-regulated simultaneously in the intestinal tract and liver of mice. Elevated expression of pro-inflammatory cytokines IL-6 and IL-1β were found in both inflamed colon tissues and the liver. Since intestinal inflammation is usually associated with bacterial dysregulation, the adherent invasive *Escherichia coli* (AIEC) strain prototype LF82 was used as an inflammatory agent. The results of co-culture with intestinal cells and liver cells were also analyzed, where FXR expression was decreased but returned to normal after LF82 removal. These observations imply that the expression of bile acid receptors reinforces the relationship between the gut and the liver (Negroni et al., 2020). Similarly, Baitouweng Tang, a traditional Chinese herbal medicine, promoted the levels of hepatic FXR and GPBAR1 to improve the relative abundance of intestinal microorganisms such as *Escherichia coli* and *Proteus* and inhibited the activity of NF-κB to treat UC mice (Hua et al., 2021).

There is an imbalance in the intestinal fungal community in IBD and the abundance of *Candida spp* is high in stool cultures of IBD patients. After a large-scale analysis, it was found that the human intestinal fungus *Candida metapsilosis M2006B* and its metabolites could specifically activate FXR, which has a good therapeutic effect on colitis and has been verified in mouse models. Therefore, *Candida metapsilosis M2006B* may be a beneficial intestinal fungus for the treatment and prevention of IBD (Huo et al., 2022). In other studies, HFDs have been found to boost bile acid production and serve as a contributing factor to UC. For example, Zhao et al. demonstrated that HFD reduced FXR expression through the transforming growth factor-β (TGF-β) pathway, thereby increasing the incidence of UC. FexD, an FXR agonist, significantly increased TGF-β signaling, upregulated TGF-β receptor 1 (TGF-β R1) and Smad2, and effectively improved disease activity index scores and weight loss in HFD and DSS-induced UC mice. On this basis, the use of TGF-β R1 inhibitor SB431542 counteracts the effects of FexD, therefore, the activation of FXR may be considered an effective target for the treatment of UC (Zhao et al., 2020).

The occurrence of IBD is closely related to the destruction of the epithelial barrier. Studies have shown that the existence of intestinal bile acids has a certain relationship with the intestinal barrier. Since primary bile acids are transformed into secondary bile acids when transported to the intestinal lumen, Mroz et al. studied DCA and UDCA-two of the most common colon bile acids, in relation to the intestinal barrier. It was found that DCA as low as 25 μM increased the expression of FXR, but the increase of FXR also inhibited the repair of colon epithelial T_84_ cells. Boyden chamber was employed to observe the migration ability of cells and it was found that the migration ability was reduced after DCA treatment. Similar to DCA, upregulation of FXR by GW4064 significantly reduced wound closure and T_84_ cell migration in the Boyden chamber. Although both activate FXR, GW4064 and DCA do not superimpose but inhibit the activity of cystic fibrosis transmembrane conductance regulator (CFTR), which is essential for colon epithelial repair, thus inhibiting wound closure and reducing migration of intestinal epithelial cells. In contrast, UDCA treatment promoted colon epithelial repair in both cell and animal models, and prevented the harmful effects of DCA, suggesting that UDCA has a beneficial effect on IBD treatment. However, the specific mechanism is not yet clear (Mroz et al., 2018). Therefore, although FXR agonists are continuously being developed and applied, their treatment of diseases may not always bring good outcomes. In other words, the regulatory activity of FXR brings different effects, therefore, it is necessary to use FXR agonists with caution.

## Therapeutic effects of FXR in colorectal cancer

As the third most general malignant tumor in the world, CRC owns an extremely high death rate (Bray et al., 2018). Risk factors include HFDs, abnormal bile acid metabolism, and disrupted gut microbiota (Miyazaki et al., 2021; Ocvirk and O'Keefe, 2021; Ocvirk et al., 2020). In human colon cancer samples, immunohistochemical (IHC) results show that there are fewer FXR markers in stage II adenocarcinoma compared to the normal colon. Moreover, mRNA levels of FXR are reduced several times in stages I, II, and III of colorectal adenocarcinoma, and are down-regulated in a stage-dependent manner, signifying that the silencing starts at the transcriptional level (Bailey et al., 2014). In several rodent models of tumorigenesis, FXR deficiency generates an obvious elevation in the size and number of colon tumors. Thus, FXR could provide targets for the prevention and treatment of CRC (Miyazaki et al., 2021).

The activation of epithelial-mesenchymal transformation (EMT) is a pivotal step in the transformation of colon cancer cells. At this stage, epithelial cells gain mesenchymal cell traits, enhancing the motility and migration of cancer cells. A primary identity of EMT is the downregulation of E-cadherin, which is associated with a poor prognosis of CRC (Vu and Datta, 2017). Yu et al. found that the number of lung metastasis nodules in HT-29-shFXR and Caco-2-shFXR was higher than the controls, along with decreased E-cadherin in HT-29-shFXR and Caco-2-shFXR cells but increased vimentin levels compared to control cells. The same observation was made in lung metastases in FXR KO BALB/C mice. Meanwhile, WNT signaling is involved in the development of EMT and FXR inhibits the WNT/β-catenin signaling activity of colon cancer cells. Activation of the WNT signaling gives rise to the increased expression of β-catenin in the cells, while overexpression of FXR can form a complex with β-catenin that disrupts the stability of the β-catenin/transcription factor 4 (TCF4) complex, thereby suppressing the transcriptional activity of WNT-related target genes. Conversely, it also antagonizes the FXR/RXRα complex and its transcriptional activity. SHP has also been shown to play a partial tumor-suppressive role, because FXR-mediated transcriptional activation of SHP inhibits the expression of cyclin D1 and C-C motif chemokine ligand 2 (CCL2), reducing the proliferation and invasion of tumor cells (Yu et al., 2020a).

**FIGURE 3:**
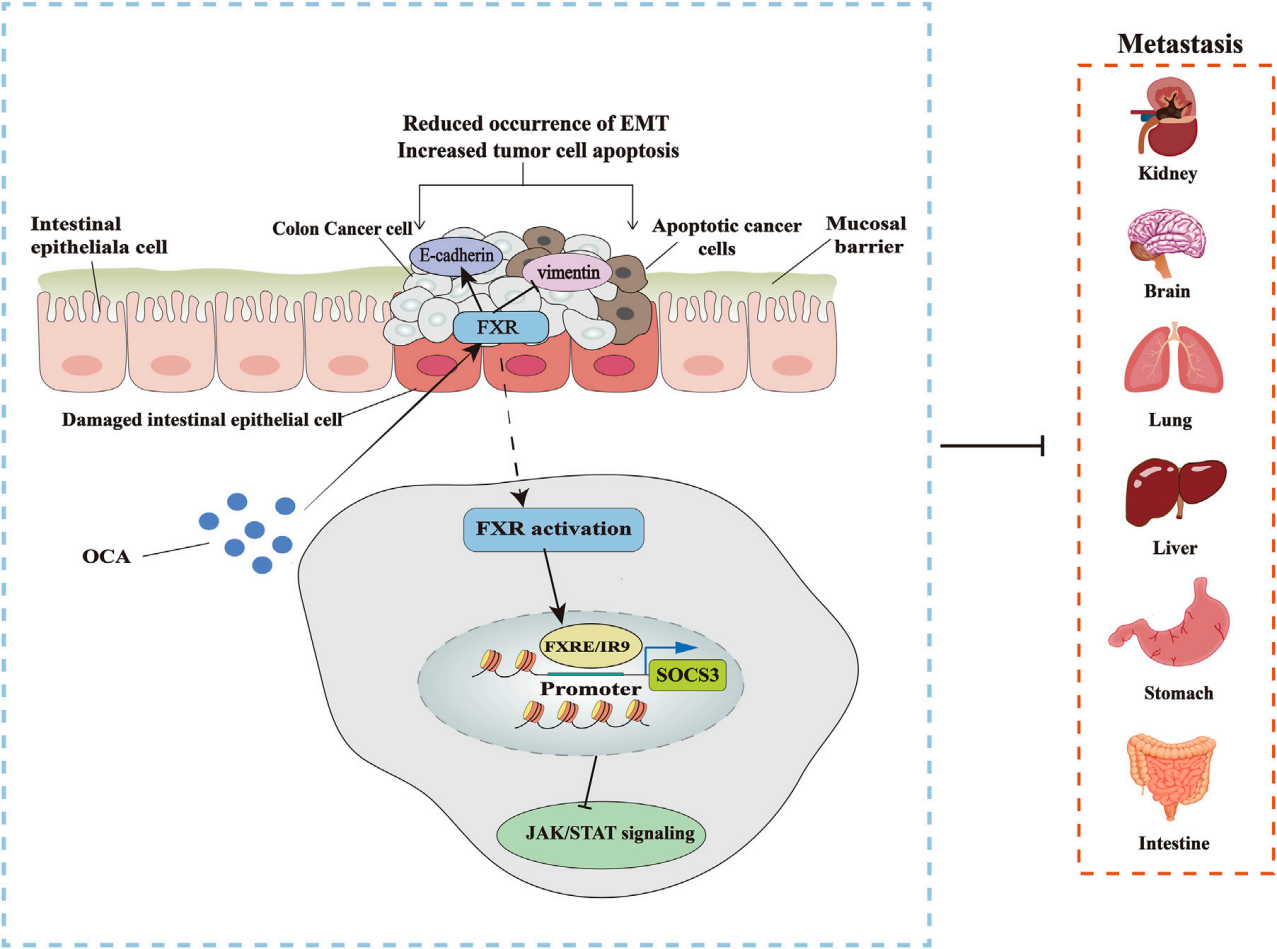
Targeting FXR to treat CRC. OCA inhibits the occurrence of EMT by activating FXR, thus blocking the metastasis of colon cancer cells and promoting the apoptosis of cancer cells, increasing the expression of EMT marker gene E-cadherin, and decreasing the level of vimentin. The specific mechanism is that OCA promotes the binding of FXR to SOCS3 promoter FXRE/IR9, inhibiting the JAK2/STAT3 pathway associated with colon cancer.

As an agonist of FXR, OCA is nearly one hundred times more potent than the natural ligand of FXR, CDCA. OCA inhibits the activity and growth of HT-29 and Caco-2, delaying the proliferation of colon cancer cells by inhibiting G1/S transition and inducing apoptosis. EMT is also inhibited during the same process. The activation of FXR in an OCA-treated group restrained the development of xenograft tumors in nude mice in comparison to a dimethyl sulfoxide (DMSO) group. Immunohistochemical staining also revealed markers in xenograft tumors, weak Ki67 expression, but a strong caspase-3 expression. It has been shown that the activity of the Janus tyrosine kinases/signal and activator of transcription (JAK/STAT) pathway is strongly associated with colon cancer, where the suppressor of cytokine signaling-3 (SOCS3) negatively regulates the JAK/STAT pathway. OCA activation of FXR upregulates SOCS3 expression at the mRNA and protein levels and inhibits the JAK2/STAT3 pathway (Figure 3) (Li et al., 2020).

However, OCA is mainly used as a treatment for liver cancer and cholangiocarcinoma in previous studies. Due to the heterogeneity of tumors, its role in CRC may not have a good tumor inhibition effect. Recent studies have found that β-catenin interacts with FXR to affect the anti-tumor effect of OCA on CRC cells. Although OCA can increase the level of FXR in CRC cells, only RKO and HCT116 cells are the most sensitive to OCA, with a significant nuclear localization of FXR, while SW403, SW480, DLD-1, and HT-29 cells exhibit medium or mild nuclear localization. The expression of β-catenin in these four cells was also markedly higher than those in RKO and HCT116. By removing β-catenin expression, nuclear FXR was significantly elevated at 2 h after OCA treatment, whereas similar levels were seen at 6 h in control cells, leading to enhanced binding of FXR to SHP promoter. It is the level of β-catenin rather than its transcriptional activity that influences the antitumor effect of OCA. OCA was used to treat CRC in combination with nitazoxanide (NTZ), an antiparasitic agent that eliminates β-catenin expression. The average combination index (CI) showed strong synergistic inhibition of β-catenin in SW403, SW480, DLD-1, and HT-29 cells to better prevent the growth of cancer cells. Compared with single drug use, the combination of the two drugs elevated the apoptosis rate and the percentage of G0/G1 phase cells but reduced the percentage of S phase cells, and also impaired the invasion ability of the cancer cells. *In vivo* experiments also demonstrated that xenograft tumors in nude mice grew more slowly, smaller, and lighter under the influence of the combination of OCA and NTZ. In summary, the combined use of OCA and NTZ is more effective against CRC. NTZ also makes up for the deficiency of OCA to some extent (Yu et al., 2021).

Downstream targets of FXR include miRNAs that have a strong influence on the development of colon cancer. MiR-135A1 is highly produced in colon cancer specimens along with cell lines, while the level of the clinical significance of cyclin G2 (CCNG2), which can inhibit cell proliferation and promote apoptosis, is negatively correlated with miR-135A1 in human CRC tissues. Bioinformatics methods predicted and verified that CCNG2 is a downstream gene of miR-135A1. Since FXR is downregulated in CRC, the activation of FXR by GW4064 induces CCNG2 expression in a miR-135A1-dependent manner *in vitro* to hinder cell cycle progression and growth of colon cancer cells. Thus, the FXR/miR-135A1/CCNG2 axis could be a crucial treatment target for CRC (Qiao et al., 2018). Oxidative stress is the result of the imbalance between antioxidant and oxidant systems in the body, and the imbalance between the two has been linked to colon cancer (Basak et al., 2020). FXR can reduce reactive oxygen species (ROS) levels and act as an antioxidant (Dong et al., 2021). Nelumal A, a novel FXR agonist, attenuates oxidative stress by upregulating local antioxidant enzymes (including catalase, glutathione peroxidase, and superoxide dismutase) to inhibit azoxymethane (AOM)/DSS-induced CRC (Miyazaki et al., 2021).

Because cancer cells are prone to relapse and traditional chemotherapy has bad effects, innovative treatments are imminently needed to prolong survival rates for CRC patients. A large number of studies have shown that FXR is a potent inhibitor of tumor development and many FXR agonists can improve the activity of FXR, Table 3 summarizes the current studies about FXR agonists on CRC and IBD. Perhaps, starting from the molecular mechanism of its involvement in CRC pathogenesis could produce a good therapeutic outcome.

**TABLE 3:** Effects of FXR agonists on IBD and CRC.

Disease	FXR agonist	Effect	Model	Reference
IBD	OCA (INT-747; 6-ECDCA)	Immune dysfunction ↓	2,4,6-trinitrobenzene sulfonic acid (TNBS) or DSS -induced colitis mice	Vavassori et al., 2009 (Vavassori et al., 2009)
		Inflammation ↓		
		Splenic DCs ↑	DSS-treated mice	Massafra et al., 2016 (Massafra et al., 2016)
		Migration of DCs and effector T cells to the colon ↓		
		Migration of Tregs to the colon↑		
		IL-10 ↑		
		Colonic inflammation ↓		
	GW4064	LPS-induced TLR4/MyD88 signaling pathway ↓	WT and FXR KO mice	Liu et al., 2017 (Liu et al., 2017)
		Barrier damage in the ileum ↓		
		Mitochondrial dysfunction ↓		
	FexD	TGF-β Signaling ↑	DSS-induced UC mice	Zhao et al., 2020 (Zhao et al., 2020)
		The phosphorylated level of Smad2 ↑		
		Tissue Damage ↓		
	Fexaramine	The bacterial abundance of short-chain fatty acids (SCFA) ↑	DCA-treated mice	Xu et al., 2021 (Xu et al., 2021)
		DCA-induced intestinal inflammation ↓		
		Intestinal FgF15 ↑		
		Bile acid synthesis ↓		
	Nelumal A	Incidence of colonic mucosal ulcers ↓	DSS-induced colitis mice	Miyazaki et al., 2021 (Miyazaki et al., 2021)
		Oxidative damage ↓		
		TNF-α ↓		
CRC	OCA (INT-747; 6-ECDCA)	SOCS3 ↑	HT-29 and Caco-2 cells; nude mice	Li et al., 2020 (Li et al., 2020)
		The activity of the JAK2/STAT3 pathway ↓		
		EMT ↓		
	Nelumal A	Incidence of adenocarcinoma ↓	AOM/DSS-induced CRC mice	Miyazaki et al., 2021 (Miyazaki et al., 2021)
		Oxidative damage ↓		
		Apoptotic cancer cells ↑		
		TNF-α ↓		

## Conclusions and future perspectives

As a receptor of bile acid metabolism, FXR has been mainly focused on liver diseases in the past, but its role in the intestine has begun to be uncovered on account of the action of enterohepatic circulation. FXR exists in immune cells, therefore, FXR has a key regulatory role in the immune system although such studies regarding IBD and CRC are very scarce. When intestinal inflammation or tumor occurs, bile acid metabolism is disturbed and immune cells increase in infiltration, thus, research on FXR and immune cells in intestinal diseases is worth further exploration. In future studies, attention should be given to the relationship between the changes in intestinal FXR level and immune cells in the course of IBD and CRC, exploring the specific mechanisms involved. At the same time, it is necessary to consider whether changes in FXR levels expressed by immune cells themselves are related to intestinal inflammation and tumorigenesis. Although the role of FXR in the intestinal tract has been emphasized due to the influence of bile acids through enterohepatic circulation, previous studies have mainly focused on the treatment of intestinal diseases with FXR agonists. There is still a large gap in the study of IBD and related CRC regarding the regulation of FXR activity by PTMs. It would be interesting to investigate whether the PTM of FXR changes in intestinal diseases. Since there are many types of PTMs, the development of drugs targeting the significant PTM changes in FXR during the development of diseases may play a more precise therapeutic effect. It is also important to note that FXR agonists are not all good due to their tissue heterogeneity and have different effects on different diseases (Girisa et al., 2021). Currently, many drugs have been found to up-regulate FXR (Lu et al., 2021), therefore, it is worth exploring whether they can be used in combination with FXR agonists to enhance the therapeutic effects of FXR agonists on IBD and CRC, or whether they are not superimposable.

At present, the use of FXR agonists in clinical trials in patients with IBD and CRC is rare, although more promising results have been documented in cell and animal studies. However, since PSC is often associated with the development of IBD and has a high risk of progressing to CRC (Indriolo and Ravelli, 2014), results of clinical trials of FXR agonists in PSC could produce therapeutic ideas for IBD and CRC. Trauner et al. conducted a 12-week double-blind randomized phase II study of the nonsteroidal FXR agonist cilofexor for the treatment of PSC patients, where 60% of the screened PSC were associated with IBD. The results showed that cilofexor had certain level of safety and tolerability. Compared with the placebo group, the patients receiving 100 mg cilofexor had significantly lower levels of ALP, ALT, AST, and gamma-glutamyltransferase (GGT), and inhibited the activity of CYP7A1 to reduce bile acid synthesis. Both profibrotic and inflammatory factors were suppressed, but itch continued to develop in 36% of patients. Importantly, none of the patients who underwent the 12-week study had worsening IBD symptoms and no new IBD patients or symptoms were developed. Regardless, the risk of cardiovascular associated conditions remains to be explored (Trauner et al., 2019). In a phase II study of OCA, more than 50% of the PSC patients had IBD symptoms. OCA had a dose-dependent ALP reduction with or without UDCA but was associated with the occurrence of severe pruritus symptoms, where patients who received OCA 5–10 mg had significantly worse pruritus symptoms (Kowdley et al., 2020). In addition, during a 14-day treatment with 60 µg/day of the FXR agonist tropifexor, patients with primary bile acid diarrhea had increased expression of FGF19, inhibited bile acid synthesis, and slower overall colonic transport without causing pruritus (Camilleri et al., 2020). These observations indicate the clinical potential of FXR and bile acids in inflammatory-associated conditions; however, several challenges, including adverse events such as pruritis remain to be overcome. The dose-dependent pruritus associated with OCA may be related to the promiscuous activation of GPBAR1 by OCA (Sepe et al., 2018), as the activation of GPBAR1 can promote the excitation of sensory neurons that transmit itch and pain (Alemi et al., 2013).

Among the bile acid receptor family, FXR and GPBAR1 are the most characterized. GPBAR1 is also expressed in the liver and intestine and has certain anti-inflammatory effects. The use of a dual FXR/GPBAR1 ligand can be somewhat superior to the use of a single agonist (Fiorucci et al., 2022). The main dual FXR/GPBAR1 ligands studied so far are BAR502 and INT-767. BAR502, which primarily prioritized GPBAR1, significantly reduces liver fibrosis and improves metabolism by promoting fat browning in preclinical studies in non-alcoholic steatohepatitis (NASH) model mice. BAR502 induces an increase in the levels of FGF15, SHP, and glucagon-like peptide 1 (GLP-1) in the intestine, effectively inhibits the synthesis of bile acids and increases insulin sensitivity in mice (Carino et al., 2017). INT-767 mainly focuses on activating FXR, particularly, hepatic FXR but not the intestinal FXR. Compared with OCA, a lower dose of INT-767 shows more effect on liver steatosis, inflammation, and fibrosis in NASH model mice. However, INT-767 has a weaker ability to activate FGF15 in the ileum compared with OCA (Roth et al., 2018). In addition, GPBAR1 is also expressed in monocytes and macrophages. A study showed that INT-767 mainly focuses on activating GPBAR1 in macrophages to inhibit LPS-induced inflammation rather than activating FXR (Miyazaki-Anzai et al., 2014). The presence of GPBAR1 in macrophages can also inhibit the activation of NLRP3 inflammasome and promote the polarization of M2 macrophages (Shi et al., 2020). However, dual FXR/GPBAR1 ligands have been rarely studied in IBD and CRC, and are of great value in future exploration.
